# Study of the effect of band gap and photoluminescence on biological properties of polyaniline/CdS QD nanocomposites based on natural polymer

**DOI:** 10.1038/s41598-020-80038-1

**Published:** 2021-01-21

**Authors:** Azita Alipour, Moslem Mansour Lakouraj, Hamed Tashakkorian

**Affiliations:** 1 Polymer Laboratory, Organic Chemistry Department, Faculty of Chemistry, University of Mazandaran, Babolsar, 47416-13534 Iran; 2 Cellular and Molecular Biology Research Center (CMBRC),, Babol University of Medical Sciences, Babolsar, 47176-47745 Iran

**Keywords:** Chemistry, Materials science

## Abstract

In this work, band gap, photoluminescence and biological properties of new bionanocomposites based on polyaniline (PANi)/hydrolyzed pectin (HPEc)/cadmium sulfide (CdS) QD nanoparticles (NPs) were studied. In order to improve the morphology and properties, CdS NPs were modified with epichlorohydrin to obtain the modified CdS (mCdS). The CdS@HPEc-g-PANi and mCdS@HPEc-g-PANi samples were synthesized via heterogeneous chemical polymerization and characterized by FTIR, ^1^HNMR, SEM/XRD, *EDX/TEM/EDX-mapping *and TGA analyses. The objective of this work is the study of physical, optical and cytotoxicity properties of the nanocomposites and comparison between them. The SEM, XRD and TGA images showed that the modification of NPs resulted in homogeneous morphology, increase of crystalline structure and high thermal stability which influenced on physical and biological property. According to UV-DRS analysis, the mCdS@HPEc-g-PANi indicated lower energy gap compared to the CdS@HPEc-g-PANi nancomposite. The presence of conductive polymer and synergy effect between the PANi and CdS caused higher PL intensity in the CdS@HPEc-g-PANi nanocomposite compared to pure CdS. The emission intensity in the mCdS@HPEc-g-PANi nanocomposite was reduced since the organic modifying agent cause reducing emission intensity. The mCdS@HPEc-g-PANi nanocomposite, due to more compatibility of organic agent with cellular walls of biological cells that help to the diffusion of metal CdS NPs into cell tissue indicated more toxicity effect on cell growth.

## Introduction

In recent years, nano-conductive polymer composites due to their electronic, various morphology, optical and biocompatibility properties have been developed as promising candidates for bio-applications such as cytotoxicity^1–10^. Among conducting polymers, nano-structured polyanilines have attracted much attention due to a lot of advantages, availability, facile preparation, maneuverability of particle size and morphology, biocompatibility, high chemical stability and good electrical conductivity^3,6,10–21^. However, practical use of polyaniline has been limited because of its low solubility, processability and weak mechanical properties. Copolymerization of polyaniline with polysaccharides has been recognized as an effective strategy to eliminate these shortcomings and favorable physicochemical properties. In recent years, polyaniline/biopolymer composites have been investigated to produce eco-friendly and high performance materials with biological properties^10–24^. One of the operative biological properties of the conductive polymers is cytotoxicity which is depended on some physicochemical features such as shape, size, surface area, agglomeration state, surface charge, elemental composition and surface activity of the material^23,24^. It was proved that the nature of nanomaterials can play an important role in cellular uptake and evaluation of toxicity aspects in conductive polymer composites.

In the other hand, QD nanoparticles with unique optical and electrical properties provide bright, highly stable and size-dependent properties that suggest high efficiency of these particles in different applications. Besides these benefits, the NPs can damage to human health and the environment. The studies showed that QDs due to size-tunable photoluminescence, electrical conductivity can act as cytotoxicitic agent in biological systems. The mechanism of action of QDs can be via production of reactive oxygen radicals, catalyst activity, and DNA damage. Also QDs with their fluorescence feature influence on cytotoxicity activity. QDs because of their and ability of formation of free radicals and easy of functionalization indicates a great potential for treatment and analysis of cancer and for drug deliver Among metal semiconductor nanoparticles, cadmium sulfide has been known as toxic compound which affect on cellular stability via inhibition of cellular growth and generation of free radicals. At the cellular level, cadmium by depletion of endogenous antioxidants prompts oxidative stress leads to cells.

However, chemically synthesized QDs can not be applied to biochemical applications owing to the insolubility in water, thereby the synthesis of organic modified QDs have been extensively investigated^23,24^. Pectin as a natural polymer with creation of a amphiphilic nature and good interfacial feature in the nanocomposite can insert CdS NPs inside itself, and whilst increase cytotoxicity, generate uniform distribution of nanoparticles in the polymer matrix. Consequently, we decided herein to prepare a new amphiphilic copolymer of polyaniline with pectin polysaccharide and uniform bionanocomposite with CdS NPs. The electrical properties such as band gap determination, and photoluminescence together with cytotoxicity of the nanocomposites were investigated.

## Experimental

### Materials and methods

Aniline was acquired from Merck (Schuchardt Germany), distilled doubly to purify and stored in a refrigerator before use. Pectin was purchased from Aldrich Company. Other compounds, including APS, epichlorohydrine, cadmium nitrate (Cd (NO_3_)_2_), sodium sulfide (Na_2_S), paratoluenesulfonic acid (PTSA), HCl, NaOH and toluene were prepared from Merck.

The X-ray diffraction patterns (XRD) were recorded using a Philips PW1730 X-ray diffractometer (Netherlands) at a scan rate of 10 °C/min. The SEM and EDX analyses were carried out by a FESEM TESCAN MIRA ∏ scanning electron microscope (Czech). The (fourier-transform infrared) FTIR spectra were attained in the range of 400–4000 cm^−1^ by Nicollet IMPACT 400 D FTIR spectrometer as KBr tablets. The TGA results were achieved by TGA-DTA METTLER TGA/STTA 851 thermogravimetry (Switzerland) under air atmosphere at a heating rate of 10 °C/min at 25 °C-600 °C.

The ***HPEc/PANi/CdS QD ***(CdS@HPEc-g-PANi) nanocomposite was prepared via cocondensation of CdS NPs during polymerization of HPEc on PANi. Initially, pectin was hydrolyzed in basic medium by NaOH solution. For this purpose, firstly, the pectin was hydrolyzed via alkaline NaOH aqueous solution (3 M) for 72 h under at 45 °C to completely de-estrification of pectin to carboxyl groups, then the product was neutralized with HCl solution. The PANi/HPEc grafted copolymer was synthesized by in situ polymerization of aniline on aqueous solution of HPEc in the presence of APS as initiator. The reaction was performed under stirring and N_2_ atmosphere. In a typical synthesis, in a three necked flask equipped with a magnetic stirrer and trap of inert gas, 1 g HPEc was dissolved in 80 mL of distilled water at 50 °C. was prepared by dissolving 1.0 g of HPEc in 80 mL of distilled water at 50 °C. The solution was kept while stirred under N_2_ (g) atmosphere, then pre-cooled APS aqueous solution with molar ration of (1:1) was slowly added into the HPEc solution to create HPEc radicals. Primarily, the solution was degassed with nitrogen, and then after 30 min about 2.0 g of aniline monomer was dissolved in 50 ml of HCl (1 M), and added dropwise into the solution. The nanocomposite was prepared by adding the aqueous solutions of cadmium nitrate and sodium sulfide into the polymerization reactants under sonication and the reaction was allowed for 24 h under inert gas and subsequently the green dark products were separated through filtering, washed with distilled water and acetone and dried in an oven at 40 °C.

**Figure 1:**
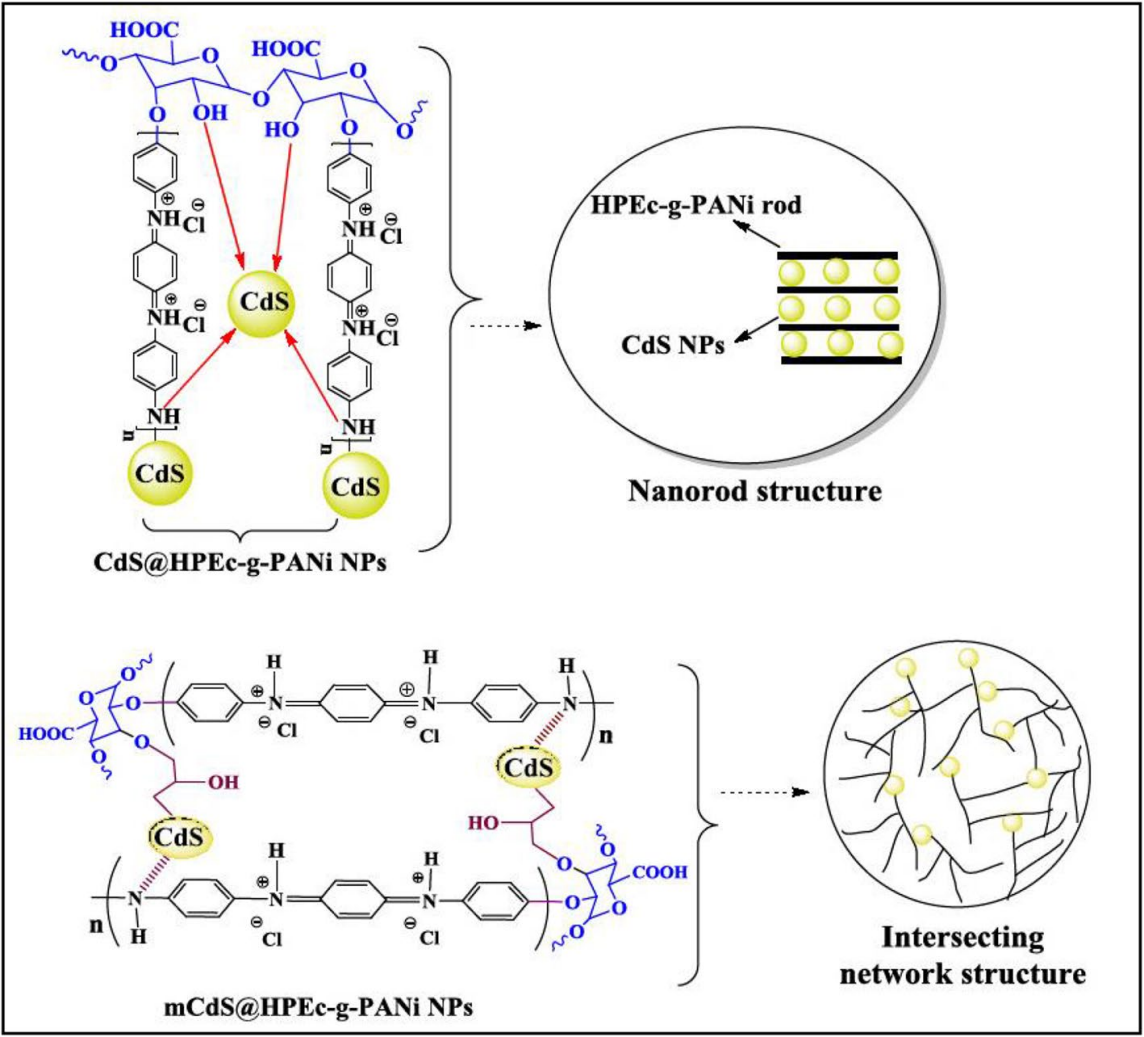
The schematic representation of the nanocomposites.

To prepare ***HPEc/PANi/mCdS QD nanocomposite*** (mCdS@HPEc-g-PANi), the CdS NPs were modified with epichlorohydrin as an organic modifying agent or capping agent. Initially the CdS NPs were prepared via mixing of aqueous solutions of Cd(NO_3_)_2_ and sodium sulfide (Na_2_S) under inert gas at 50 °C. The aqueous medium was converted to yellow color, immediately due to the formation of CdS. The stirring was continued until a certain time for completing the formation of the NPs. The precipitates were separated by high speed centrifuge and washed with water and acetone to remove unreacted and byproduct species. Finally the precipitates were dried at 60 °C overnight. In the next step, CdS NPs was dispersed in 25 ml toluene under sonication then 1 ml of epichlorohydrin was added to the reaction mixture and the reaction was continued for 72 h stirring at reflux temperature under N_2_ atmosphere. The resulting product was washed with distilled water and acetone and dried in an oven at 40 °C. In the continue, HPEc was chemical stabilized on mCdS via adding PTSA as acid catalyst, for 24 h at 70 °C. The resulting precipitates were separated via centrifugation and washed with water and dried in an oven in 50 °C. Then, the PANi was synthesized via polymerization of 1 ml of aniline in 25 cc HCl (1 M) on HPEc/mCdS NPs in the presence of APS. The polymerization mixture was continued under N_2_ gas at 50 °C. The resulted product was centrifuged and dried in an oven at 50 °C. The schematic presentation of the nanocomposites was shown in Fig. 1.

MTT test was performed on Fibroblast, HT29 and PC3 cells. MTT (3-[4,5-dimethylthiazole-2-yl]-2,5 diphenyltetrazolium bromide) is oxidized by mitochondrial dehydrogenase in living cells and gives a dark purple formazan product. Damaged or dead cells show little or no dehydrogenases activity. The experiments were conducted with a standard concentration and different sample concentrations of 62.5, 125, 250 and 500 μg/ml at 48 and 72 h incubation time. The cytotoxicity measurements were recorded as cell growth relative to untreated control cells and the results were reported as cell viability %:

$${\text{Cell viability }}\% \, = {\text{ X}}/{\text{X}}_{{\text{c}}} \times 100\%$$

where X is the cell growth amount in a particular polymer concentration and Xc is the cell growth amount for untreated control cells.

## Results and discussion

### FT-IR analysis

**Figure 2:**
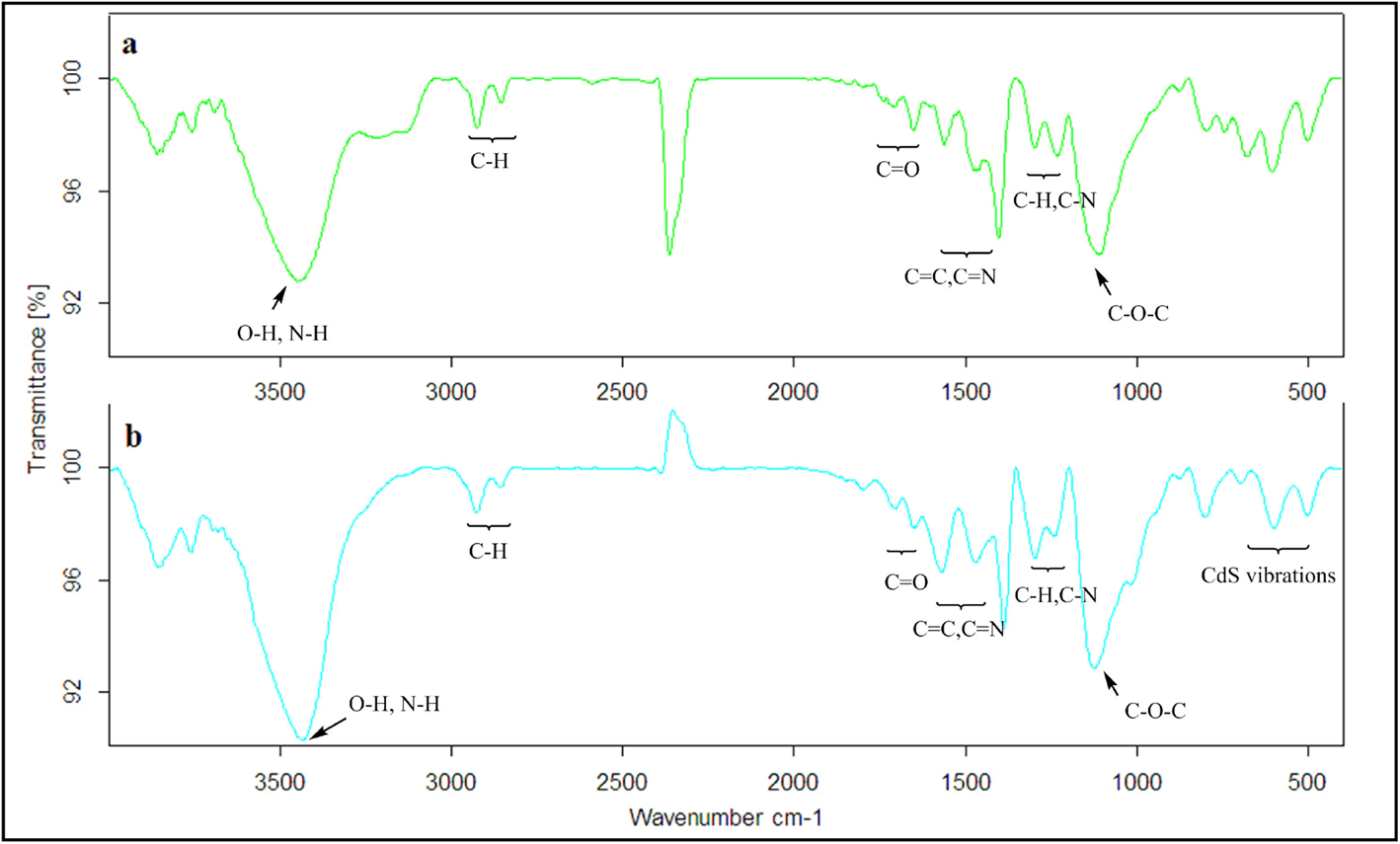
The IR spectra of (**a**) HPEc-g-PANi copolymer and (**b**) CdS@HPEc-g-PANi nanocomposite.

**Figure 3:**
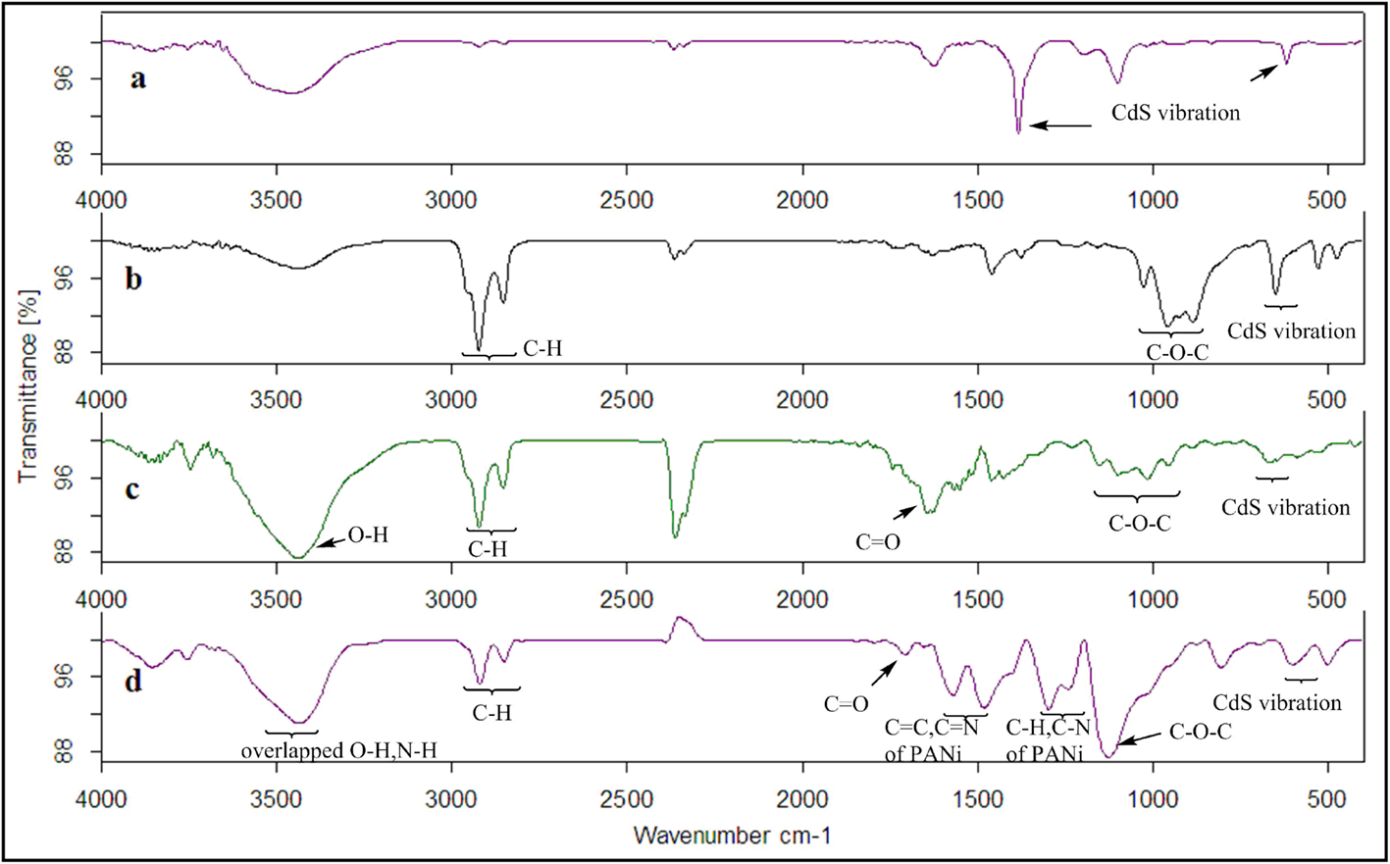
The IR spectra of (**a**) CdS NPs, (**b**) mCdS NPs, (**c**) mCdS@HPEc NPs, (**d**) mCdS@HPEc-g-PANi nanocomposite.

The FTIR spectra of the samples were shown in Figs. 2 and 3. For better comparison, the FTIR spectrum of the samples was compared to the grafted HPEc-g-PANi copolymer. In the FTIR spectra of HPEc-g-PANi copolymer, the absorption bands of PANi appeared around ~ 1474, ~ 1561, ~ 1232, ~ 1298, ~ 797 cm^−1^ attributed to the C=C and C=N vibration modes of benzenoid and quinoid unites, the bending vibration of C–H and C–N stretching of benzenoid structure and out of plane C–H vibration of benzenoid unit of PANi respectively^26–28^. The peaks of ~ 3448, ~ 2854, ~ 2923, ~ 1110 and ~ 1652, ~ 1707 cm^−1^ are corresponded to O–H, C–H aliphatic, C–O–C and C=O stretching vibrations of HPEc. The vibrational bands of CdS NPs appeared at ~ 502, ~ 599 and ~ 698 cm^−1^. In the FTIR of the CdS@HPEc-g-PANi nanocomposite, the C=C stretching vibration of benzeoid band in PANi decreased compared to HPEc-g-PANi due to the presence of CdS and electrostatic dπ-pπ interactions between Cd and N atoms that cause reduced vibrations. In FTIR spectra of Fig. 3a, the bands of ~ 1625, ~ 1384, ~ 1100 and ~ 619 cm^−1^ are attributed to the CdS vibrations^29,30^. In the FTIR spectrum of the modified CdS NPs (mCds) (Fig. 3b), the clear peaks appeared at ~ 886–1027 cm^−1^ and ~ 2854, 2924 cm^−1^ that are attributed to C–O–C and aliphatic C-H vibration which suggesting successful modification of CdS NPs. The characteristic peaks of HPEc and PANi appeared at the related area that indicative the successful formation of the nanocomposite.

### ^***1***^***H NMR analysis***

**Figure 4:**
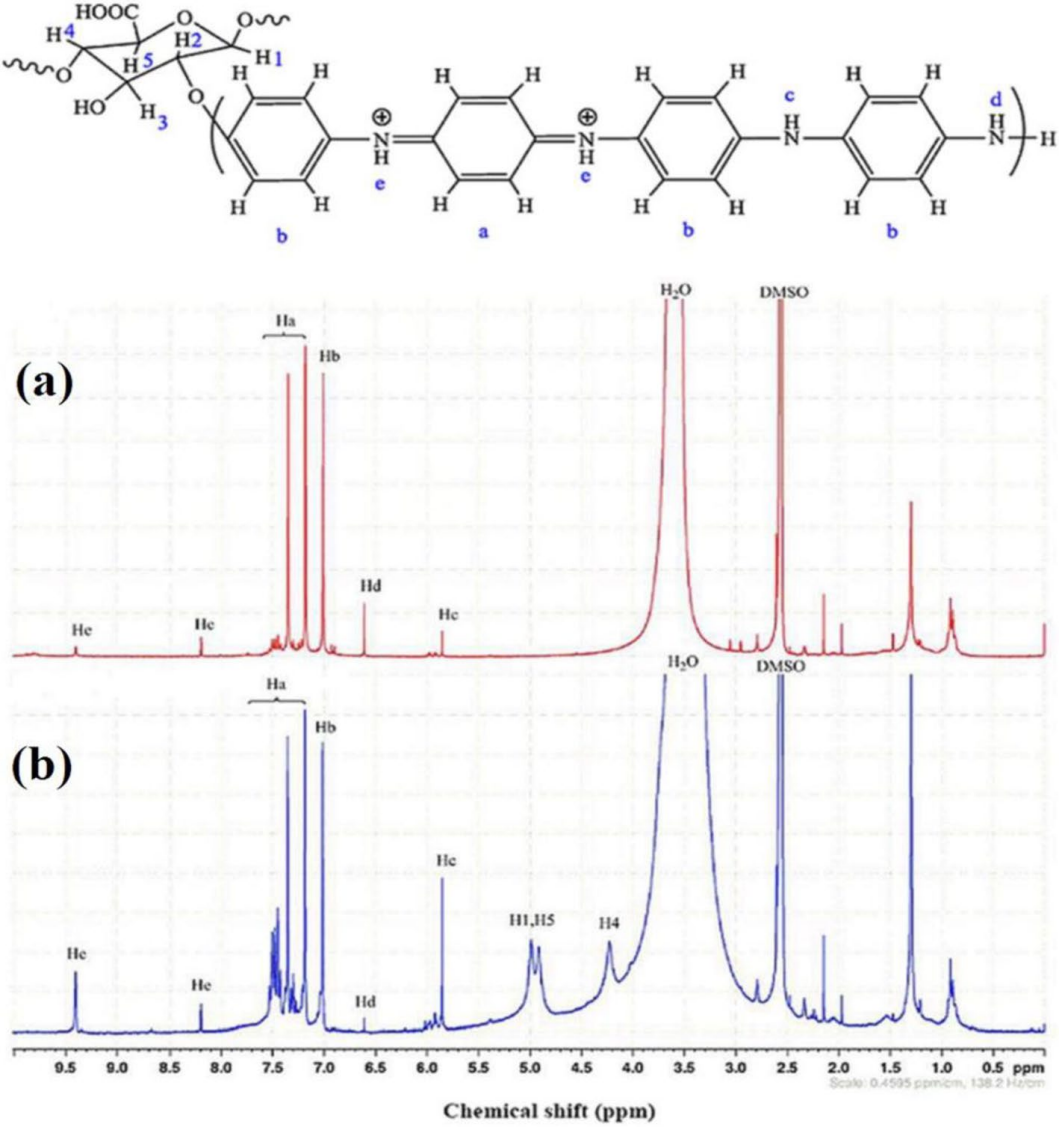
^1^H NMR spectra of PANi (**a**) and HPEc-g-PANi (**b**) in DMSO-d_6_.

For determination of grafting linkage of HPEc on PANi in the CdS@HPEc-g-PANi composite, ^1^H NMR spectra of PANi and HPEc-g-PANi was performed. The ^1^H NMR spectrum of PANi (Fig. 4a) showed characteristic peaks at δ = 6 and 6.5 ppm corresponding to –NH and –NH_2_ protons attached to the benzene rings, at δ = 7 and 7.2–7.5 ppm assigned to the protons of aromatic rings and at δ = 8.2 and 9.5 ppm ascribed to –NH^+^ protons of the quinonoid units of PANi. HPEc-g-PANi copolymer (Fig. 4b) indicated the same characteristic peaks at δ = 7–7.5 ppm attributed to protons on aromatic rings, δ = 6 and 6.5 ppm related to –NH and –NH_2_ protons on benzenoid units, and δ = 8.2 and 9.5 ppm attributed to –NH^+^ protons on quinonoid units respectively. Additionally, new peaks were detected at δ = 4.3, 4.9 and 5 ppm assigned to H4, H5 and H1 of HPEc structural units respectively^31–33^ that confirm the successful formation of the grafted copolymer.

### SEM/XRD analysis

**Figure 5:**
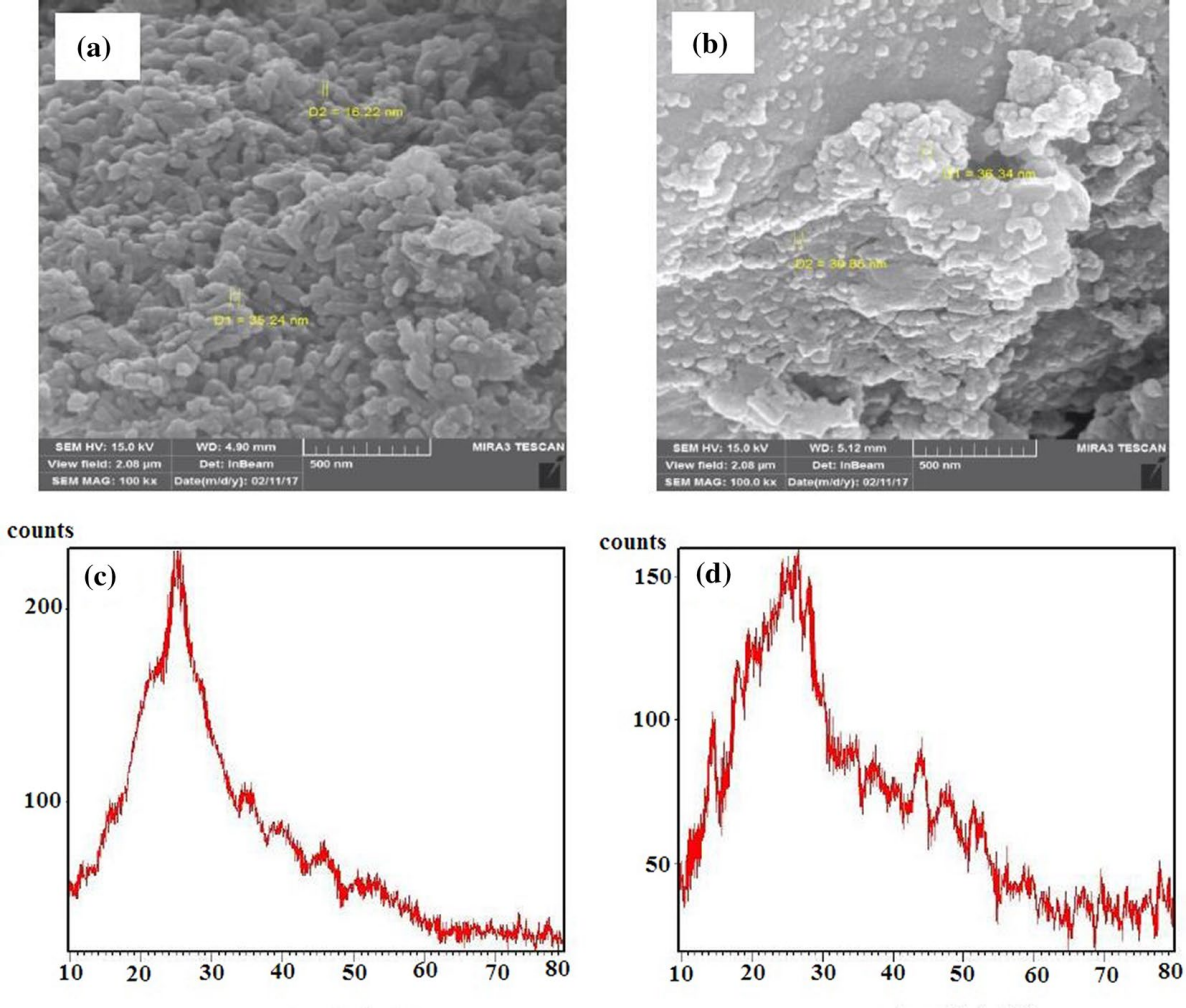
The SEM images (**A**,**B**) and XRD images (**C**,**D**) of the CdS@HPEc-g-PANi and mCdS@HPEc-g-PANi nanocomposites.

**Figure 6:**
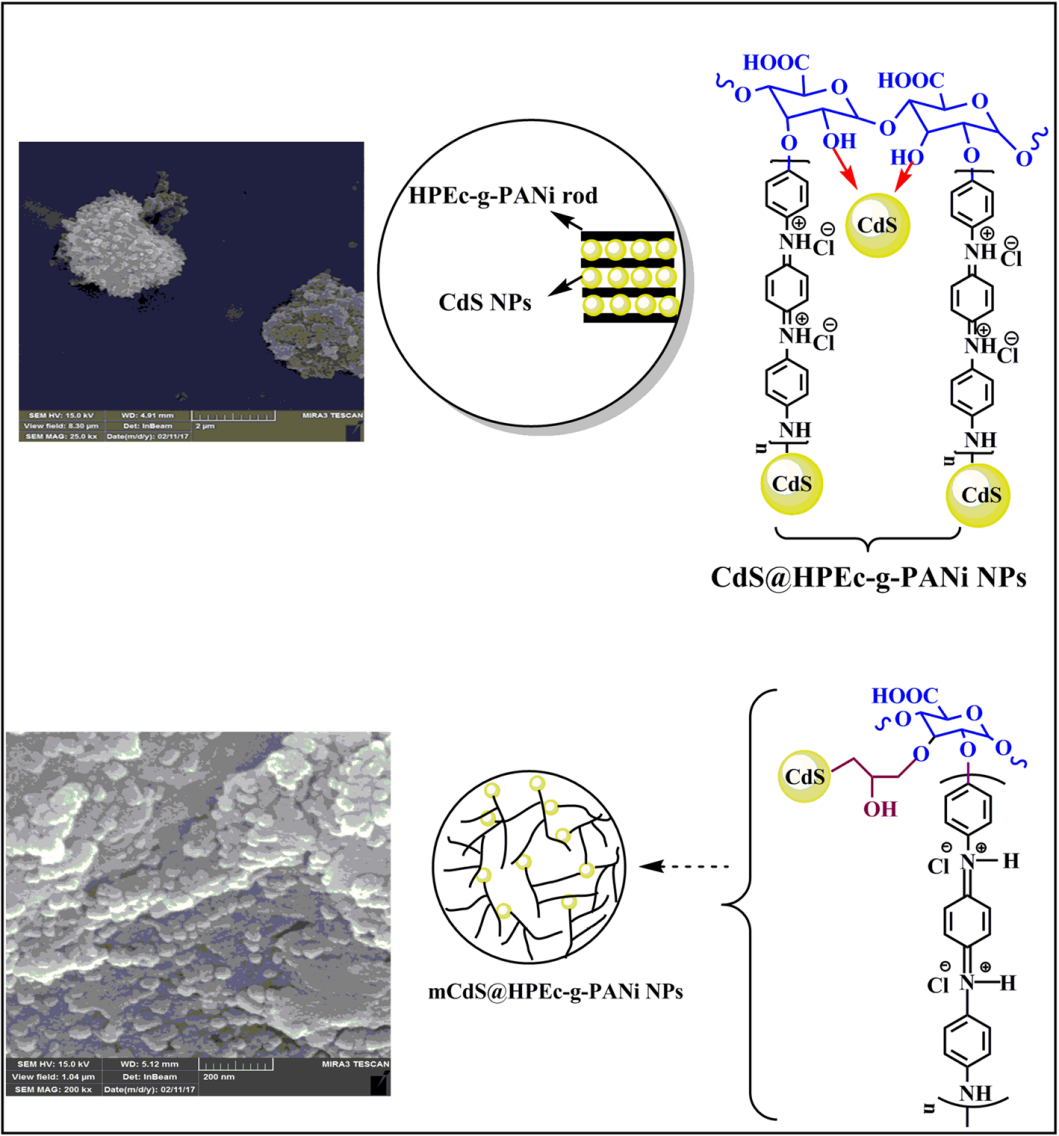
The schematic representation of synthesized nanostructures with morphology images.

The SEM analysis of surface morphology of the nanocomposites is shown in Fig. 5A,B. According to the micrographs, as shown in schemes, the mCdS@HPEc-g-PANi showed a spherical morphology and the micrograph indicated a uniform intersecting network structure because of better dispersion of the CdS NPs in the composite matrix resulted from the modification of CdS NPs. The CdS@HPEc-g-PANi nanocomposite showed a typical homogeneous rod-shape morphology. In the CdS@HPEc-g-PANi nanocoposite due to electrostatic interactions of nitrogen atoms of PANi with Cd metal centers, the polymeric macromolecules are grown as rod-shap from CdS QDs centers. The schematic representation of synthesized nanostructures with morphology images are shown in Fig. 6.

The XRD spectra of the samples were shown in Fig. 5C,D. As specified from diffraction spectra, the nanocomposites showed a typical semicrystalline morphology. The peaks at around 2θ = 25° are correspond to (110, 111) and (100) crystalline planes of PANi and CdS NPs respectively. Also the peak at 2θ = 20 °C is ascribed to (100) reflection of the crystalline plane of PANi. In the mCdS@HPEc-g-PANi nanocomposite, the small crystalline peaks were appeared in areas of 2θ = 37°, 44°, 48°, 52°, 55°, 60°, 67°–70°, 70°–80° can be corresponded to (102), (110), (103), (112), (004), (202), (203, 210), (211,114, 212) reflections of CdS NPs respectively^34,35^. The appearance of these crystalline peaks and the cohesive crystalline structure in this nanocomposite can be indicating better distribution of the CdS NPs in the matrix of nanocomposite, compared to the CdS@HPEc-g-PANi nanocomposite, which rearrangement of polymer chains and subsequently the crystallinity of PANi in composite is increased. Generally, the modified mCdS@HPEc-g-PANi nanocomposite showed more specified crystallinity relative to the CdS@HPEc-g-PANi nanocomposite due to more improved dispersion of the CdS NPs and regular crystalline structure of the polymeric chains in the nanocomposite matrix.

### EDX/TEM/EDX-mapping analysis

**Figure 7:**
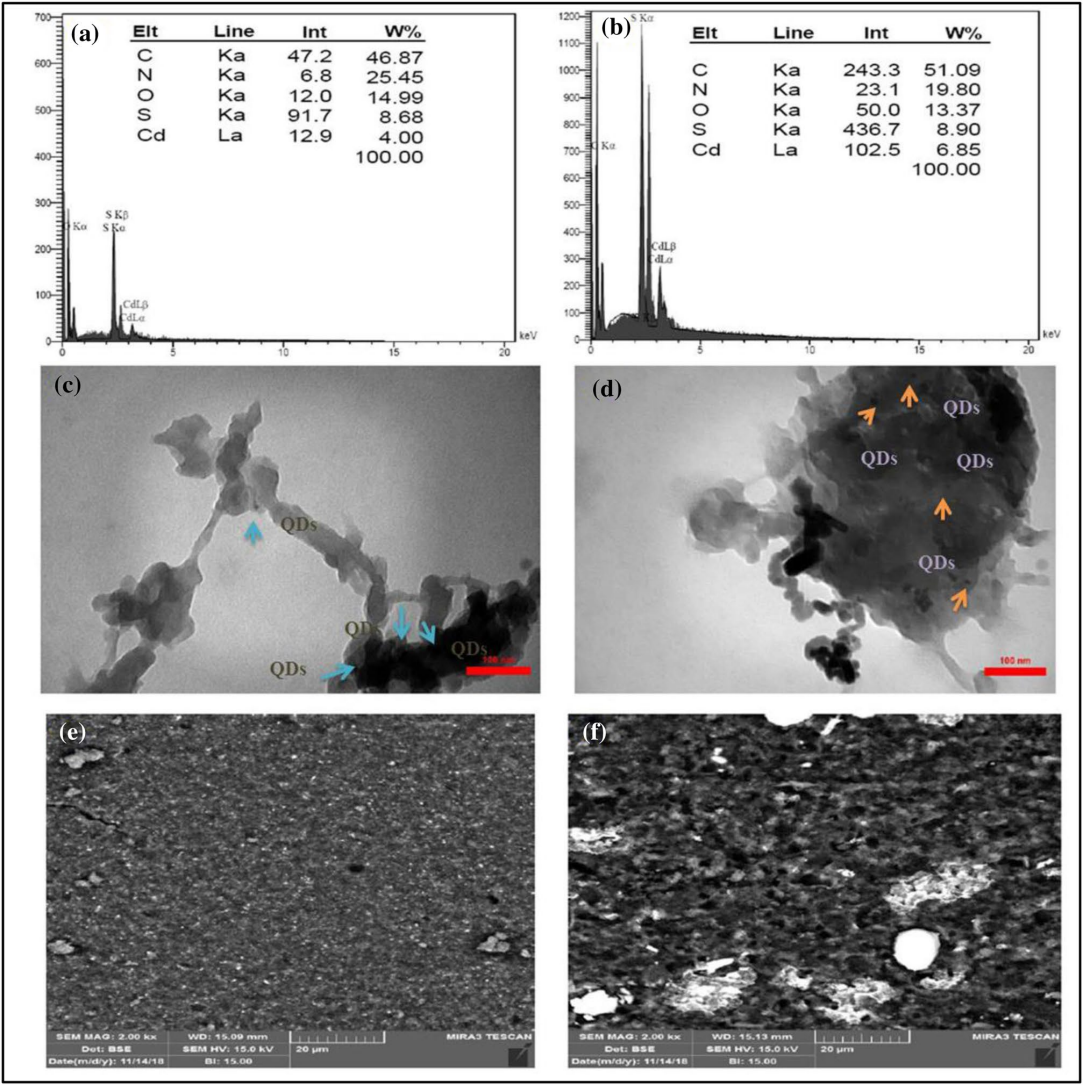
The images of EDX spectra of CdS@HPEc-g-PANi (**a**), mCdS@HPEc-g-PANi (**b**), TEM images of CdS@HPEc-g-PANi (c), mCdS@HPEc-g-PANi (**d**) and EDX-map of CdS@HPEc-g-PANIi (**e**) and mCdS@HPEc-g-PANi (**f**).

The elemental composition of the samples by EDX (Fig. 7a,b) showed the existence of elements of Cd and S and in the nanocomposites.

The internal morphology of the CdS@HPEc-g-PANi and mCdS@HPEc-g-PANi nanocomposites were identified by TEM analysis (Fig. 7c,d) to verify the decoration of CdS nanoparticles on the surface of HPEc-g-PANi copolymer. QDs were shown as dark spots on gray area of composite. The TEM images obtained showed similar morphologies with SEM images, nanorod and spherical structures of the deposition of the CdS nanoparticles in the composites.

The EDX-mapping image (Fig. 7e,f) for observation of the contribution of CdS NPs in the nanocomposites verified the homogeneous distribution of CdS NPs in the composites that mixed well with HPEc-g-PANi polymeric particles.

### TGA analysis

**Figure 8:**
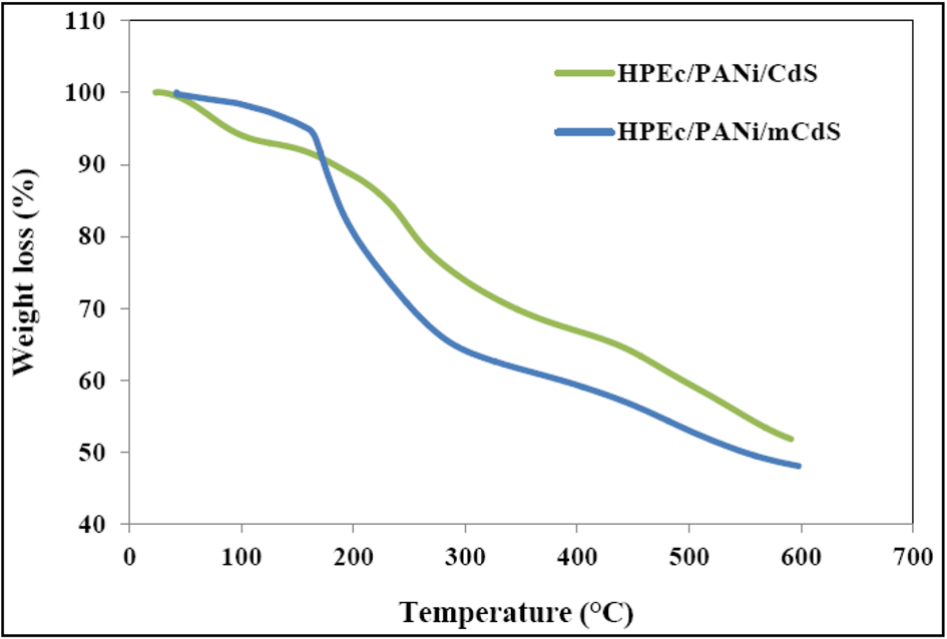
The TGA curves of the (**a**) CdS@HPEc-g-PANi and (**b**) mCdS@HPEc-g-PANi nanocomposites.

The TGA thermograms of the nanocomposites are reported in Fig. 8. The CdS@HPEc-g-PANi nanocomposite exhibited a weight loss step in 25 to 100 °C, which is consistent with the exclusion of residual water, vaporization and elimination of volatile products from the material^1,36^. The second step of weight loss was observed at 248–370 °C attributed to the decomposition of the HPEc structure, the elimination of dopant ions^1,36–39^. The third step of weight loss after 421 °C is mainly due to degradation of the polymer backbone. The TGA curve of the mCdS@HPEc-g-PANi displayed a weight loss step between 25 and 150 °C temperature range due to the elimination of water and volatile substances from the nanocomposite. A weight loss at 150–300 °C attributed to the removal of dopant molecules, and scission of the ether linkage of HPEc. The weight loss after 425 °C that is mainly assigned to the degradation of the nanocomposite. According to the results of total weight loss percentage and degradation temperature (T_d_) reported in Table 1, the modified mCdS@HPEc-g-PANi nanocomposites demonstrated relatively higher thermal stability compared to the unmodified CdS@HPEc-g-PANi nanocomposite due to better dispersion of CdS NPs. The amount of weight loss in the modified mCdS@HPEc-g-PANi nanocomposite is much higher and retention weight is lower than that of the CdS@HPEc-g-PANi nanocomposite because of the existence of soft organic modifying agent which facilitate thermal degradation.

**Table 1:** The TGA results, including T_d_ and weight loss (%) of CdS@HPEc-g-PANi and mCdS@HPEc-g-PANi nanocomposites.

Sample	Ti	T_d_ (°C)	Weight loss (%)
CdS@HPEc-g-PANi	120	421	48.14%
mCdS@HPEc-g-PANi	170	425	51.68%

### Band gap

The band gap of the samples was calculated according to the following equation (take equation):

1 $$\left( {\upalpha {\text{h}}\upnu } \right) \, = {\text{ A }}\left( {{\text{h}}\upnu - {\text{Eg}}} \right)^{{\text{n}}}$$

where α, αhʋ, A, hʋ, Eg are absorption coefficient, light frequency function, proportionality constant, photon energy and energy gap. n defines the electronic transition nature, for direct transition, n = 1/2 and for indirect transition , n = 2. Linear extrapolation of (αhʋ)^2^ versus hʋ gives the amount of band gap.

**Figure 9:**
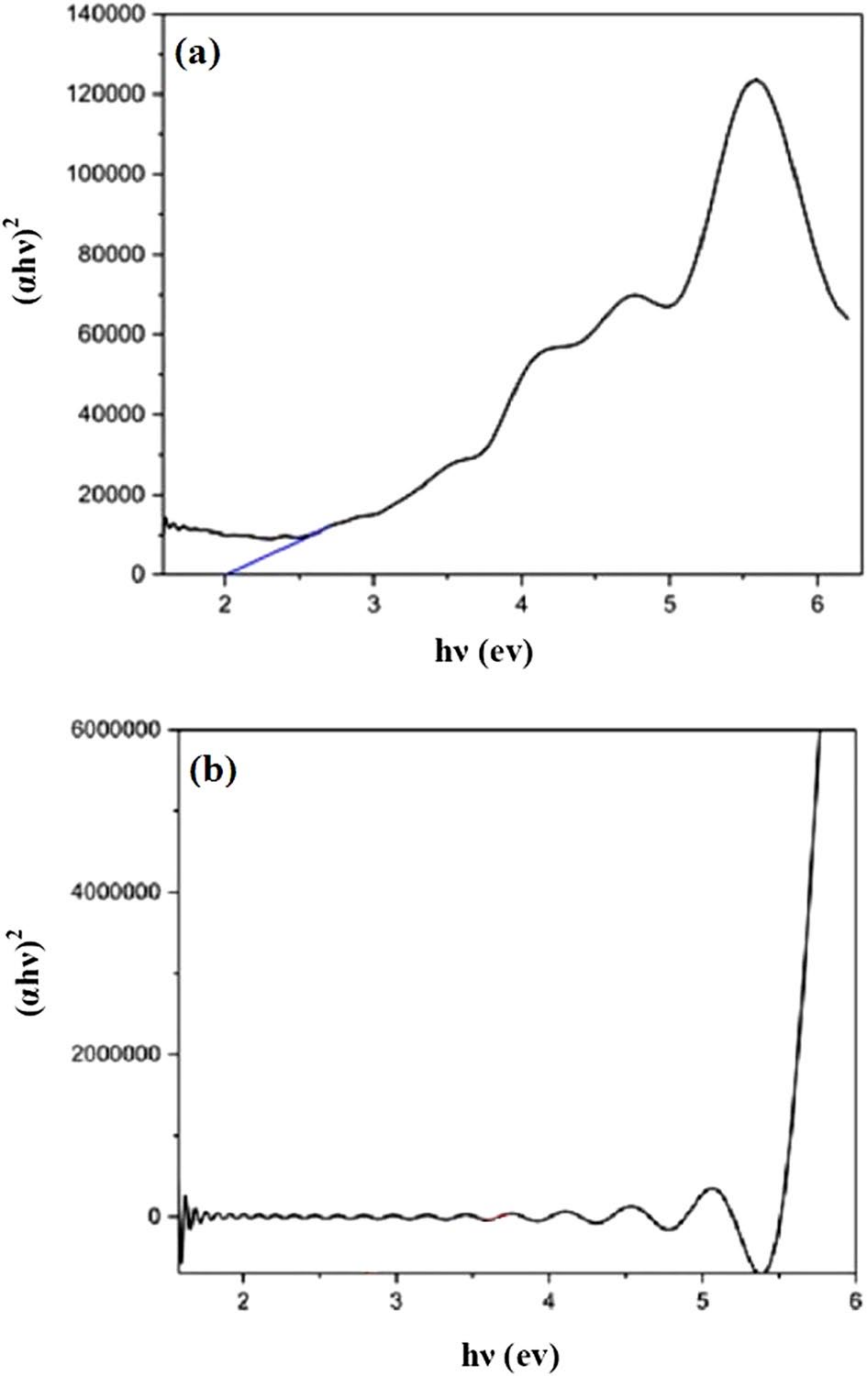
The UV-DRS curves of and (**a**) mCdS@HPEc-g-PANi and (**b**) CdS@HPEc-g-PANi nanocomposites.

The mCdS@HPEc-g-PANi nanocomposite showed lower band gap about 2 eV compared to CdS@HPEc-g-PANi nanocomposite (2.7 eV). This result is typically representative homogeneous dispersion of the CdS NPs in the nanocomposite which cause near energy gaps of the electronic levels of the CdS NPs owing to electrostatic interactions of the Cd centers with PANi active sites, and in fact, the synergy effect between components. The UV/DRS curves of the nanocomposites are shown in Fig. 9.

### Photoluminescence

**Figure 10:**
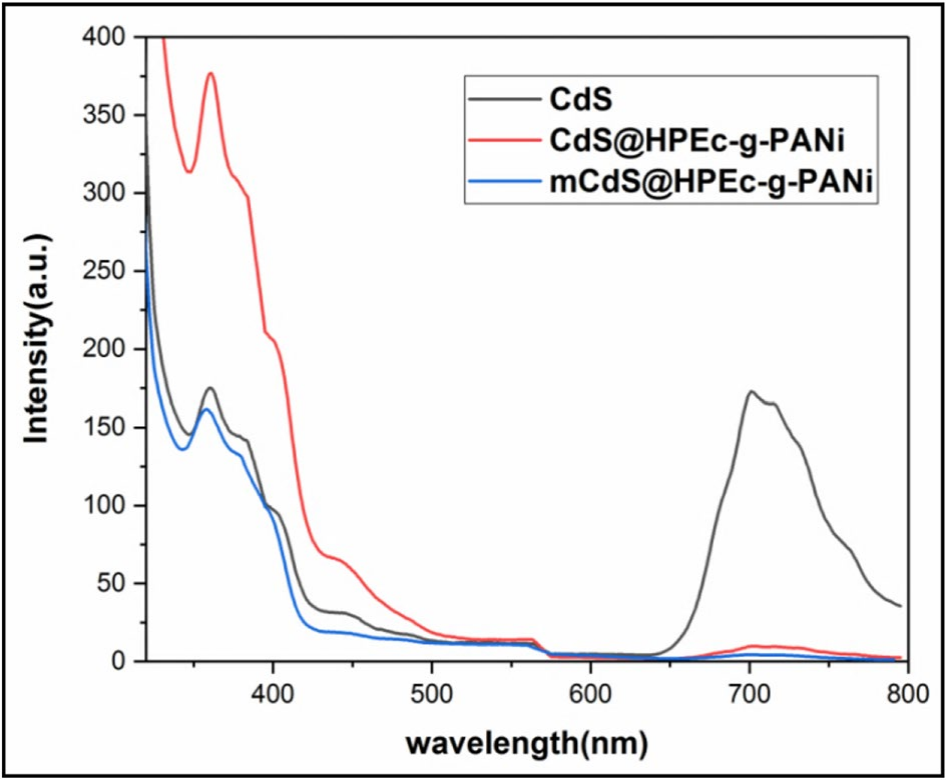
The PL spectra of the samples.

**Figure 11:**
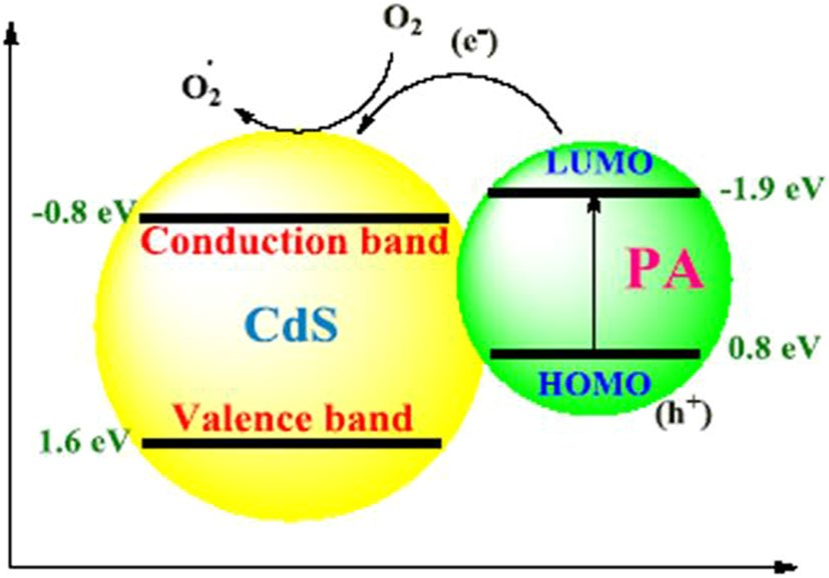
The electronic levels connection of PANi and CdS.

The PL spectra of CdS, CdS@HPEc-g-PANi and mCdS@HPEc-g-PANi in excitation wavelength of 300 nm are shown in Fig. 10. The PL spectra showed emission peaks in 370 nm and 700 nm and weak peaks at 400–500 nm attributed to band transition of CdS NPs. The peak at 500 nm and 700 nm can be attributed to band-edge emission and trap-state emission of CdS NPs. The CdS@HPEc-g-PANi nanocomposite showed a considerable increase in emission intensity compared to CdS NPs, due to strong chelating and electrostatic interaction between PANi and CdS and efficient electronic transitions. PANi with imposing of a synergy effect on energy levels of CdS NPs, and creation of a proportional electronic structure (heterojunction structure) (Fig. 11) cause reduction of band gap between the electronic levels of HOMO of PANi and valence band of CdS and LUMO of PANi and conduction band of CdS, and facilitation of excitation of the electrons and increase of emission intensity. In addition, with the formation of this electronic structural relationship and synergistic effect of PANi on CDs, electron-holes are formed on electronic levels of PANi and CdS that leads to increase of charge separation and as a result increase of PL emission intensity. The mCdS@HPEc-g-PANi nanocomposite exhibited lower intensity due to the presence of the organic modifying agent. The organic modifying agent with the creation of structural defects in the interface between CdS NPs, the creation of multiple pathways and different light trapping modes on NPs decrease PL emission intensity.

### Cytotoxicity test

**Figure 12:**
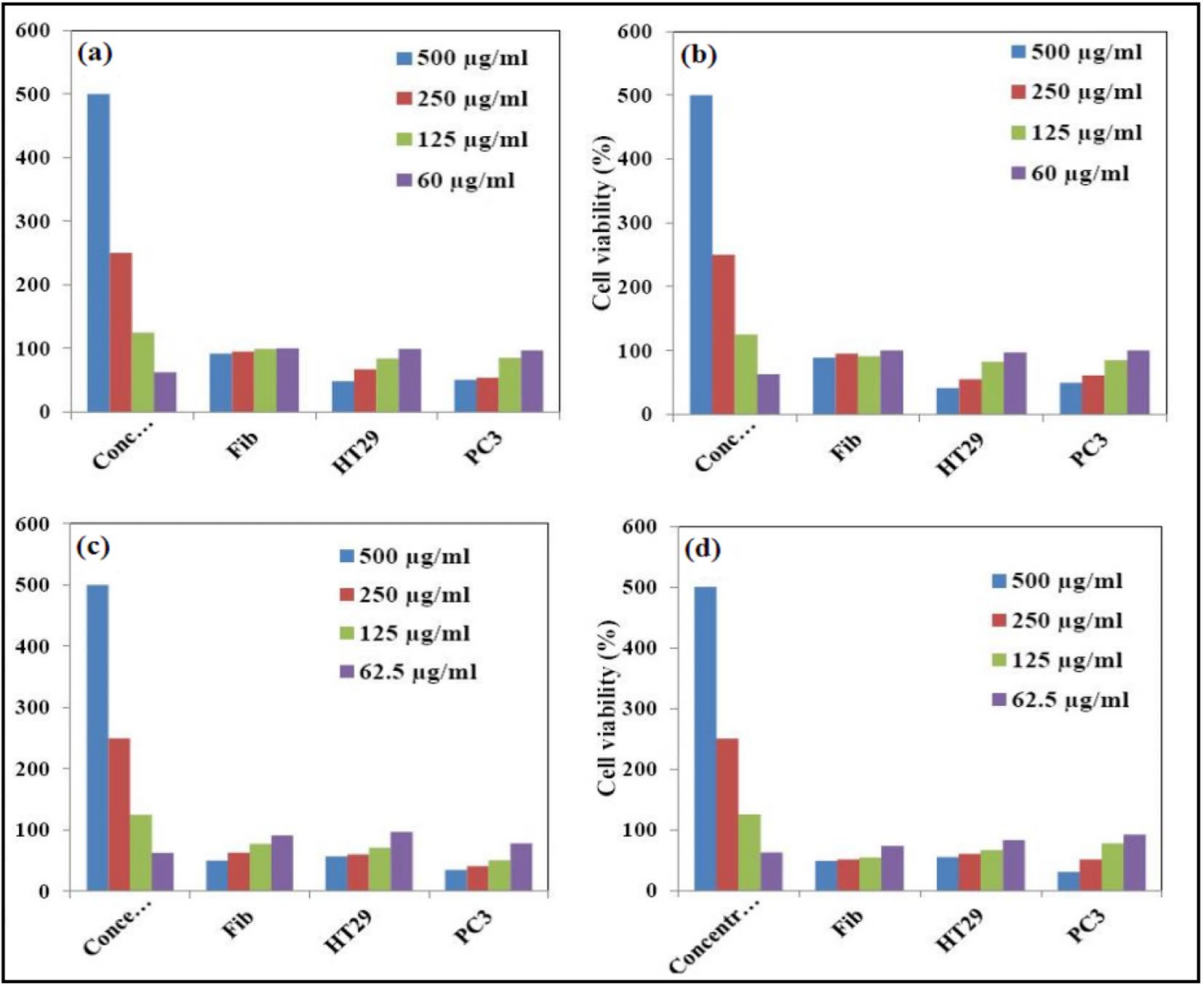
% Cell viability obtained from MTT assay for Fib, HT29 and PC3 cells treated with (**a**) CdS@HPEc-g-PANi, 48 h, (**b**) CdS@HPEc-g-PANi, 72 h, (**c**) mCdS@HPEc-g-PANi, 48 h, (**d**) mCdS@HPEc-g-PANi, 72 h incubation with the nanomaterials.

**Figure 13:**
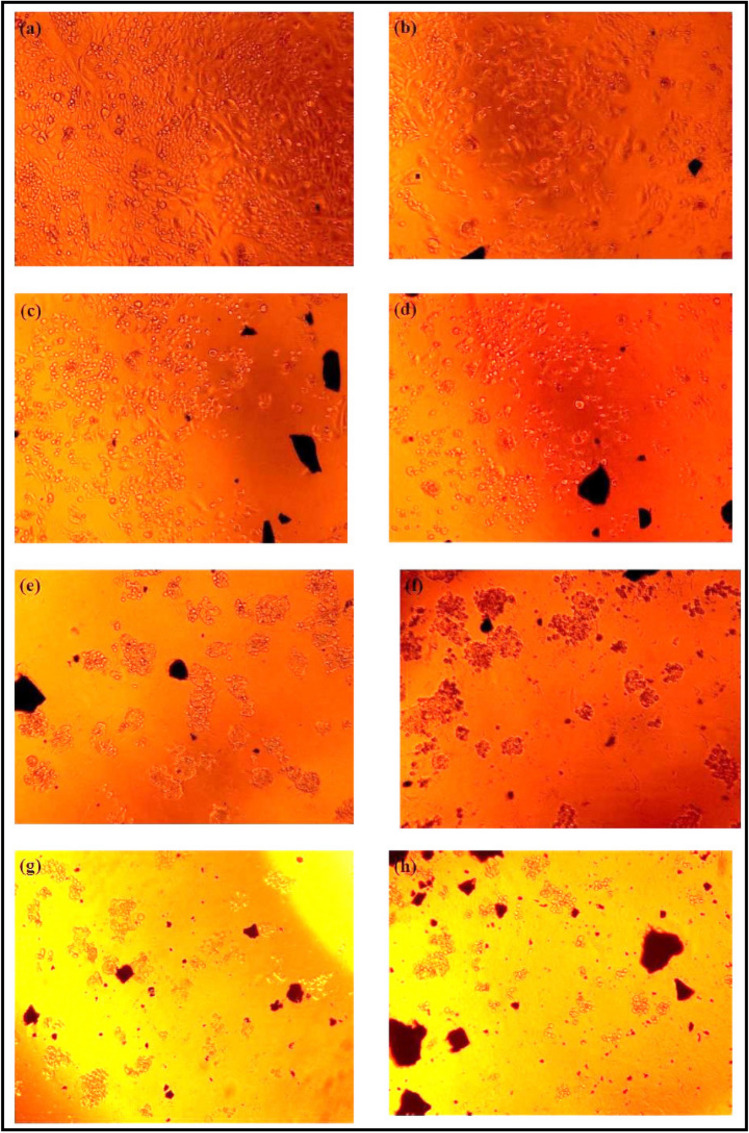
Cytotoxicity Images of mCdS@HPEc-g-PANi (**a**) (60 μg/ml, (**b**) 125 μg/ml, (**c**)250 μg/ml, (**d**) 500 μg/ml on PC3 and CdS@HPEc-g-PANi (**e**) 60 μg/ml, (**f**) 125 μg/ml, (**g**) 250 μg/ml, (**h**) 500 μg/ml on HT29.

**Figure 14:**
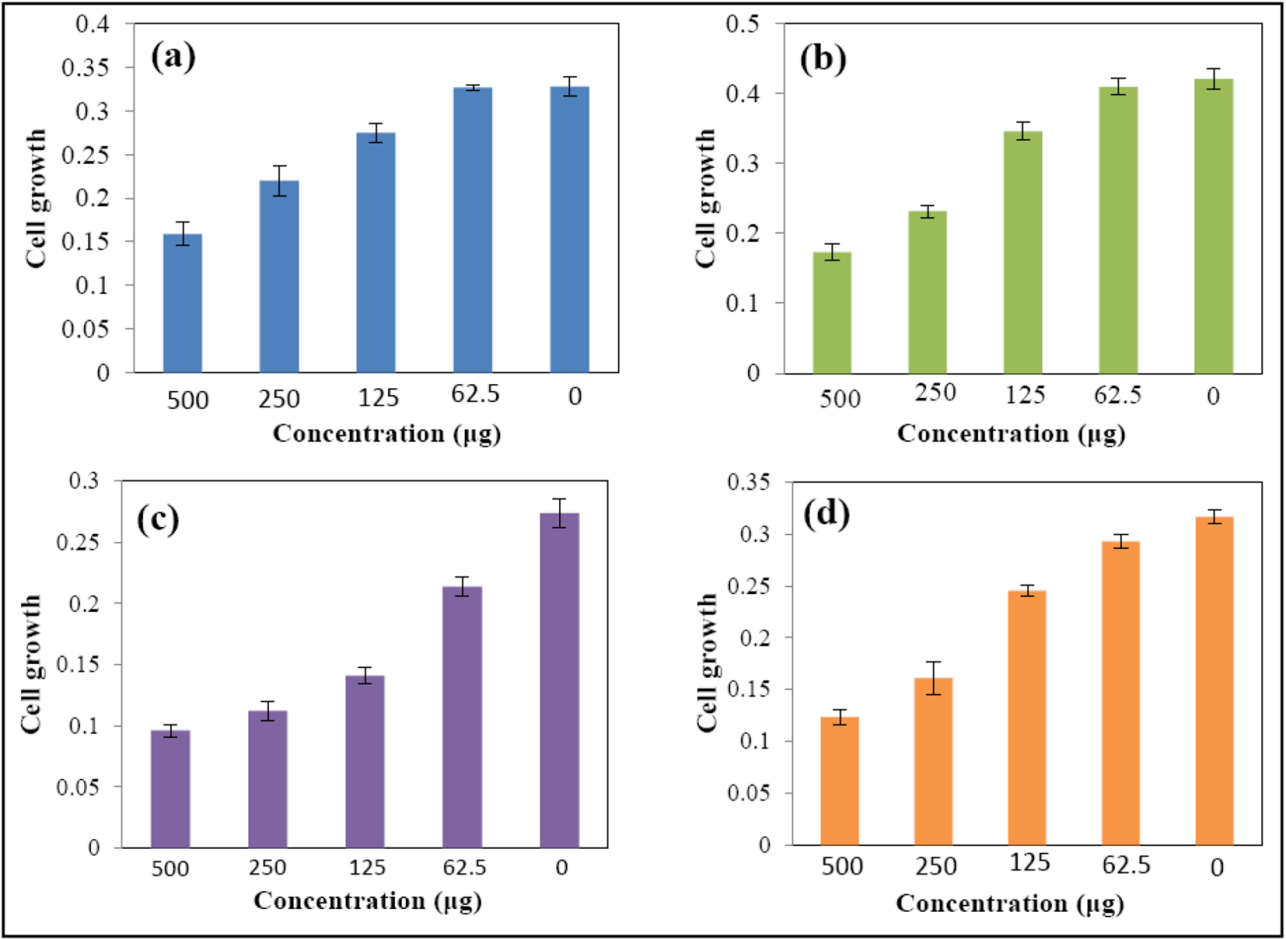
The cytotoxicity images of the nanocomposites with eror bar of the average growth of cells for the most effective cells of HT29 for the CdS@HPEc-g-PANi at 48 h (**a**) and 72 h (**b**) and PC3 for the mCdS@HPEc-g-PANi nanocomposite at 48 h (**c**) and 72 h (**d**).

The results of cytotoxicity as cell viability % and images are shown in Figs. 12 and 13. According to the results, cell viability didn’t show not much change in low concentrations, but considerably decreased in high concentration (500 μg/ml) at both 48 and 72 h. With the increase of sample concentration and incubation time, cytotoxicity reached its most optimum amount at the concentration of 500 μg/ml in 72 h. The CdS@HPEc-g-PANi showed most efficient cytotoxicity on HT29 cells and mCdS@HPEc-g-PANi most effective performance on PC3 cells. CdS NPs because of the effect of toxicity and photocatalyst activity can influence on cell growth. The mCdS@HPEc-g-PANi nanocomposite due to more homogeneous morphology and better dispersion of CdS NPs in the nanocomposite matrix showed slightly more toxicity on cells. In the other hand, the existence of organic modifying agent helps to diffuse the CdS NPs into biological cells and the toxicity effect on cells. However, the modifying agent reduces fluorescence intensity and leads to reduction of electrostatic interactions with biological cells and reduction of cytotoxicity, for this reason, the modified nanocomposite showed slightly more toxicity relative to the unmodified nanocomposite. The results of cytotoxicity were reported in Table 2. Moreover, the cell growth amounts, growth average, standard deviation and cell vitability % of the cytotoxicity results of the most effective cells, HT29 for the CdS@HPEc-g-PANi and PC3 for the mCdS@HPEc-g-PANi nanocomposites were reported in Table 3. The cytotoxicity images of the nanocomposites with eror bar of the average growth of cells for the most effective cells of HT29 for the CdS@HPEc-g-PANi and PC3 for the mCdS@HPEc-g-PANi nanocomposite were shown in Fig. 14.

**Table 2:** The cytotoxicity results of the CdS@HPEc-g-PANi and mCdS@HPEc-g-PANi nanocomposites.

	CdS@HPEc-g-PANi	Fib	HT29	PC3
48 h	500 µg/ml	0.194	0.159	0.139
	250 µg/ml	0.202	0.22	0.148
	125 µg/ml	0.209	0.275	0.234
	62.5 µg/ml	0.211	0.327	0.267
	Control	0.211	0.328	0.274
72 h	500 µg/ml	0.211	0.173	0.157
	250 µg/ml	0.226	0.232	0.192
	125 µg/ml	0.217	0.346	0.27
	62.5 µg/ml	0.237	0.41	0.319
	Control	0.237	0.421	0.317
	mCdS@HPEc-g-PANi			
48 h	500 µg/ml	0.105	0.189	0.096
	250 µg/ml	0.132	0.197	0.112
	125 µg/ml	0.162	0.234	0.141
	62.5 µg/ml	0.191	0.319	0.214
	Control	0.211	0.328	0.274
72 h	500 µg/ml	0.114	0.228	0.123
	250 µg/ml	0.121	0.247	0.161
	125 µg/ml	0.128	0.273	0.245
	62.5 µg/ml	0.173	0.345	0.293
	Control	0.237	0.415	0.317

**Table 3:** The cell growth amounts, growth average, standard deviation and cell vitability % of the cytotoxicity results of the most effective cells, HT29 for the CdS@HPEc-g-PANi and PC3 for the mCdS@HPEc-g-PANi nanocomposites.

Concentration (μg/ml)	CdS@HPEc-g-PANi			Average	Standard deviation	Cell vitability %
500	0.145	0.161	0.172	0.159	0.01358	48
250	0.209	0.219	0.242	0.22	0.01741	67
125	0.279	0.263	0.284	0.275	0.01098	84
62.5	0.325	0.326	0.331	0.327	0.00324	99
Control	0.339	0.328	0.317	0.328	0.011	100
500	0.174	0.161	0.185	0.173	0.01202	41
250	0.222	0.235	0.239	0.232	0.00889	55
125	0.334	0.359	0.346	0.346	0.01251	82
62.5	0.423	0.4	0.409	0.41	0.01162	97
Control	0.428	0.416	0.402	0.421	0.01475	100

Concentration (μg/ml)	mCdS@HPEc-g-PANi			Average	Standard deviation	Cell vitability %
500	0.098	0.100	0.09	0.096	0.00529	35
250	0.120	0.105	0.111	0.112	0.00755	41
125	0.134	0.142	0.148	0.141	0.00704	51
62.5	0.218	0.205	0.219	0.214	0.00781	78
Control	0.287	0.265	0.271	0.274	0.01138	100
500	0.121	0.118	0.131	0.123	0.00682	38
250	0.145	0.161	0.177	0.161	0.016	51
125	0.239	0.246	0.25	0.245	0.00557	77
62.5	0.298	0.297	0.286	0.293	0.00671	92
Control	0.315	0.325	0.312	0.317	0.00667	100

## Conclusion

The band gap, photoluminescence and biological properties of polyanilin/CdS/pectin nanocomposites were studied in the present work. In order to improve the morphology and properties, CdS NPs were modified with epichlorohydrin as capping agent to be dispersed homogeneously in the nanocomposite matrix. The samples were synthesized via heterogeneous chemical polymerization and characterized via different analyses of FTIR, ^1^HNMR, XRD/SEM, EDX/TEM/EDX-mapping and TGA. The SEM and XRD showed a rod-like, spherical and semicrystalline morphology for the nanocomposites. The modified mCdS@HPEc-g-PANi nanocomposite showed higher thermal stability relative to the unmodified CdS@HPEc-g-PANi nanocomposite. The unmodified CdS@HPEc-g-PANi nanocomposite due to the absence of organic modifying agent and free surfaces of CdS NPs showed higher PL intensity compared to the mCdS@HPEc-g-PANi nanocomposite. The cytotoxicity analyses were performed on Fibroblast, HT29 and PC3 cells via MTT assay. The mCdS@HPEc-g-PANi nanocomposite indicated the best toxicity effect on cells, the presence of organic modifying and better dispersion of CdS NPs helped to diffusion of CdS NPs into biologic cells and more toxicity. According to the UV-DRS analysis, due to the more homogeneous dispersion of CdS NPs, the mCdS@HPEc-g-PANi showed lower band gap compared to the CdS@HPEc-g-PANi nancomposite.
